# Supplementary material for: Mechanisms governing the pioneering and redistribution capabilities of the non-classical pioneer PU.1
Source: Nat Commun. 2020 Jan 21;11:402. doi: 10.1038/s41467-019-13960-2 (PMC6972792; doi:10.1038/s41467-019-13960-2)
Supplement: Supplementary file 7 — Source data [file 41467_2019_13960_MOESM7_ESM.zip › Source_Data/FigureS2/FigureS2B_MotifScanOutput/KmeansCluster1/homerResults/motif10.similar.html]

motif10

## Information for motif10

A
T
G
C
G
T
C
A
A
T
G
C
A
C
G
T
A
C
G
T
A
G
T
C
A
C
G
T
T
C
A
G
A
G
T
C
G
C
A
T
C
G
A
T
G
A
C
T
  
Reverse Opposite:  

C
T
G
A
C
G
T
A
C
G
T
A
A
C
T
G
A
G
T
C
C
G
T
A
A
C
T
G
C
G
T
A
C
G
T
A
T
A
C
G
A
C
G
T
A
T
C
G
  

|  |  |
| --- | --- |
| p-value: | 1e-101 |
| log p-value: | -2.332e+02 |
| Information Content per bp: | 1.802 |
| Number of Target Sequences with motif | 186.0 |
| Percentage of Target Sequences with motif | 3.65% |
| Number of Background Sequences with motif | 104.9 |
| Percentage of Background Sequences with motif | 0.26% |
| Average Position of motif in Targets | 101.7 +/- 29.0bp |
| Average Position of motif in Background | 105.1 +/- 63.6bp |
| Strand Bias (log2 ratio + to - strand density) | -0.4 |
| Multiplicity (# of sites on avg that occur together) | 1.01 |
| Motif File: | file (matrix) reverse opposite |

### Similar de novo motifs found

|  |  |  |  |  |  |  |  |
| --- | --- | --- | --- | --- | --- | --- | --- |
| Rank | Match Score | Redundant Motif | P-value | log P-value | % of Targets | % of Background | Motif file |
| 1 | 0.881 | C G T A A T G C A C G T A C G T A G T C A G C T A C T G A G T C C G A T C G T A | 1e-47 | -110.419714 | 2.97% | 0.54% | motif file (matrix) |
| 2 | 0.897 | A G T C C G T A A G T C A C G T A C G T A G T C A C G T A C T G A G T C | 1e-32 | -75.404767 | 1.79% | 0.28% | motif file (matrix) |
| 3 | 0.796 | A G T C C G T A A T G C A C G T A C G T A G T C A C G T A T G C | 1e-32 | -74.973299 | 8.13% | 4.08% | motif file (matrix) |
| 4 | 0.797 | A G T C C G T A A G T C A C G T A C G T A T G C A C G T | 1e-21 | -49.406306 | 20.47% | 15.08% | motif file (matrix) |
